# Supplementary material for: Modelling microplastic bioaccumulation and biomagnification potential in the Galápagos penguin ecosystem using Ecopath and Ecosim (EwE) with Ecotracer
Source: PLoS One. 2024 Jan 24;19(1):e0296788. doi: 10.1371/journal.pone.0296788 (PMC10807758; doi:10.1371/journal.pone.0296788)
Supplement: S1 File — (DOCX) [file pone.0296788.s001.docx]

**Supplementary Information**

**Supplementary Table S1:** Sources used to determine modelling parameters for basic Ecopath estimates.

|  | Diet | Biomass (t/km^2^) | Q/B & P/B |
| --- | --- | --- | --- |
| Galápagos penguin | [48,54,57,62,70; E. Espinoza pers. Comm., Galápagos National Park, October 2021, unpublished data] | 0.125 [54] | Q/B = 60.3  P/B = 0.067  [54] |
| Barracuda | [54] | 13.06  [54] | Q/B = 3.9  P/B = 0.063  Fishbase  Life-history: *Sphyraena idiates* |
| Mullet | [54] | 22.6 [54] | Q/B = 10.9  P/B = 2.8  Fishbase Life-history: *Mugil galapagensis* |
| Anchovy, Herring, Sardines, Salema | [54,71–73] | 19 [54] | Q/B = 15  P/B = 4.6  Fishbase  Life-history: *Brachygenys jessiae, Sardinops sagax, Anchoa nasus, Opisthonema berlangai* |
| Decapods | [54] | 14.48 [54] | Q/B = 11.95  P/B = 0.687  Sealifebase  *Scyllarides astori* |
| Predatory zooplankton | [54] | 15 [54] | Q/B = 99.13  P/B =45  [54] |
| Herbivorous zooplankton | [54] | 22 [54] | Q/B = 200  P/B = 36  [54] |
| Macroalgae | NA | 800.47 [54] | NA |
| Microalgae/  phytoplankton | NA | 31.16 [54] | NA |
| Detritus | NA | 500 [54] | NA |

**Supplementary Table S2:** Modelling scenarios and respective Ecotracer input data for model kinetic parameters.

| Scenario | Description | Initial concentration & base inflow (t/km^2^) | Decay Rate | Direct absorption rate for zooplankton  (g/kg) | Elimination rates |
| --- | --- | --- | --- | --- | --- |
| 1 | Baseline | 4.11x10^-2^ | 0.0283 | 7.38x10^-2^ | Table 5 |
| 2 | High Environmental Concentration | 7.83x10^-2^ | 0.0283 | 7.38x10^-2^ | Table 5 |
| 3 | Low Environmental Concentration | 3.9x10^-3^ | 0.0283 | 7.38x10^-2^ | Table 5 |
| 4 | Elimination rates changed to 99% eliminated in 1 day | 4.11x10^-2^ | 0.0283 | 7.38x10^-2^ | All groups set to 0.99 |

**Supplementary Table S3:** Functional group input data for the Galápagos penguin (GP) model based on the baseline scenario.

|  | Group name | Direct absorption rate | Prop. of contaminant excreted |
| --- | --- | --- | --- |
| 1 | Galápagos Penguin |  | 0.333 |
| 2 | Barracuda |  | 0.02 |
| 3 | Mullet |  | 0.03333 |
| 4 | Anchovy, Herring, Sardines, Salema |  | 0.02 |
| 5 | Decapods |  | 0.07 |
| 6 | Predatory zooplankton | 7.38x10^-7^ | 0.14 |
| 7 | Herbivores zooplankton | 7.38x10^-7^ | 0.14 |
| 8 | Macroalgea |  |  |
| 9 | Microalgea/phytoplankton |  |  |
| 10 | Detritus |  |  |

**Supplementary Table S4:** Functional group input data for the Bolivar Channel Ecosystem (BCE) model based on the baseline scenario.

|  | Group name | Direct absorption rate | Prop. of contaminant excreted |
| --- | --- | --- | --- |
| 1 | Phytoplankton |  |  |
| 2 | Macroalgae + others |  |  |
| 3 | Surgeonfishes, chubs and giant damiselfishes |  | 0.02 |
| 4 | Sea cucumbers and other |  | 0.14 |
| 5 | Herbivorous zooplankton | 7.38x10^-7^ | 0.14 |
| 6 | Sea turtles and marine iguanas |  | 0 |
| 7 | Small hervivorous gastropods |  | 0.14 |
| 8 | Sponges and polichaetes |  | 0.14 |
| 9 | Gorgonians |  | 0.14 |
| 10 | Parrotfish |  | 0.02 |
| 11 | Mullets |  | 0.033 |
| 12 | Benthic omnivorous fish |  | 0.02 |
| 13 | Anemones and zoanthids |  | 0.14 |
| 14 | Sea stars and sea urchins |  | 0.14 |
| 15 | Planktivorous reef fish (>15cm) |  | 0.02 |
| 16 | Small planktivorous reef fish |  | 0.02 |
| 17 | Lobsters |  | 0.07 |
| 18 | Predatory zooplankton | 7.38x10^-7^ | 0.14 |
| 19 | Big gastropods and other sea stars |  | 0.14 |
| 20 | Small predator gastropods |  | 0.14 |
| 21 | Benthic predatory fish (<30cm) |  | 0.02 |
| 22 | Benthic predatory fish (>30cm) |  | 0.02 |
| 23 | Barracudas |  | 0.02 |
| 24 | Groupers |  | 0.02 |
| 25 | Jacks and mackerels |  | 0.02 |
| 26 | Rays |  | 0.02 |
| 27 | Predatory marine mammals |  | 0.33 |
| 28 | Seabirds |  | 0.33 |
| 29 | Sharks |  | 0.02 |
| 30 | Detritus |  |  |

**Supplementary Table S5:** Linear regression data for the GP EwE model with the baseline scenario showing the significant relationship between predicted concentrations of microplastics (log-transformed data) and trophic levels in the GP model at years 1, 25, 50, and 100. The antilog of the regression slope was used to determine TMF (i.e., TMF= 10^b^).

| Statistical regression parameters | Year 1 | Year 25 | Year 50 | Year 100 |
| --- | --- | --- | --- | --- |
| Correlation Coefficient (r) | 0.87 | 0.93 | 0.93 | 0.92 |
| Coefficient of determination (r^2^) | 75.56% | 86% | 86% | 85% |
| Slope for least square line (b) | 0.8499 | 1.61 | 1.74 | 1.79 |
| y-intercept for least square line | -10.7613 | -11.41 | -11.54 | -11.65 |
| P-value* | 0.011 | 0.0028 | 0.0028 | 0.0032 |
| *TMF* | 7.08 | 40.7 | 55.0 | 61.7 |

*Statistically significant linear regression

**Supplementary Table S6:** Bioaccumulation factor (BAF), bioconcentration factor (BCF), and predator-prey biomagnification factors (BMF_TL_) from average microplastic concentration (g/kg) from selected predator-prey combinations in the Galápagos penguin food web model.

|  | Predatory zooplankton with prey set to herbivorous zooplankton | Planktivorous fish with prey set to predatory zooplankton | Planktivorous fish with prey set to herbivorous zooplankton | Mullet with prey set to detritus | Galápagos penguin with prey set to planktivorous fish | Galápagos penguin with prey set to mullet | Galápagos penguin with prey set to decapods |
| --- | --- | --- | --- | --- | --- | --- | --- |
| BAF | 4.36x10^-8^ | 4.92x10^-8^ | 4.92x10^-8^ | 2.26x10^-8^ | 5.90x10^-5^ | 5.90x10^-5^ | 5.90x10^-5^ |
| BCF | 4.36x10^-8^ | 4.92x10^-8^ | 4.92x10^-8^ | 2.26x10^-8^ | 5.90x10^-5^ | 5.90x10^-5^ | 5.90x10^-5^ |
| BMF_TL_ | 3.50 | 6.84 | 3.06 | 11.3 | 1239 | 1796 | 2308 |

**Supplementary Table S7:** Linear regression data for the BCE with the baseline scenario showing the significant relationship between predicted concentrations of microplastics (log-transformed data) and trophic levels in the GP model at years 1, 25, 50, and 100. The antilog of the regression slope was used to determine TMF.

| Statistical regression parameters | Year 1 | Year 25 | Year 50 | Year 100 |
| --- | --- | --- | --- | --- |
| Correlation Coefficient (r) | 0.62 | 0.79 | 0.79 | 0.79 |
| Coefficient of determination (r^2^) | 38.83% | 62.65% | 61.87% | 61.87% |
| Slope for least square line (b) | 0.80 | 1.20 | 1.22 | 1.22 |
| y-intercept for least square line | -11.1 | -10.7 | -10.6 | -10.6 |
| P-value* | 0.0005 | 8.8x10^-7^ | 1.15x10^-6^ | 1.148x10^-6^ |
| *TMF* | 6.17 | 15.8 | 16.6 | 16.6 |

*Statistically significant linear regression

**Supplementary Table S8:** Bioaccumulation factor (BAF), bioconcentration factor (BCF), and predator-prey biomagnification factors (BMF_TL_) from average microplastic concentration (g/kg) from selected predator-prey combinations in the BCE web model.

|  | Predatory zooplankton with prey set to herbivorous zooplankton | Planktivorous fish with prey set to predatory zooplankton | Planktivorous fish with prey set to herbivorous zooplankton | Mullet with prey set to detritus | Seabirds with prey set to planktivorous fish | Seabirds with prey set to mullet | Seabirds with prey set to lobster |
| --- | --- | --- | --- | --- | --- | --- | --- |
| BAF | 7.34x10^-8^ | 6.62 x10^-8^ | 6.62x10^-8^ | 1.14x10^-8^ | 5.85x10^-7^ | 5.85x10^-7^ | 5.85x10^-7^ |
| BCF | 7.34x10^-8^ | 6.62 x10^-8^ | 6.62x10^-8^ | 1.14x10^-8^ | 5.85x10^-7^ | 5.85x10^-7^ | 5.85x10^-7^ |
| BMF_TL_ | 4.57 | -2.73 | -2.73 | 18.3 | 8.61 | 36.9 | 75.8 |

A)

| 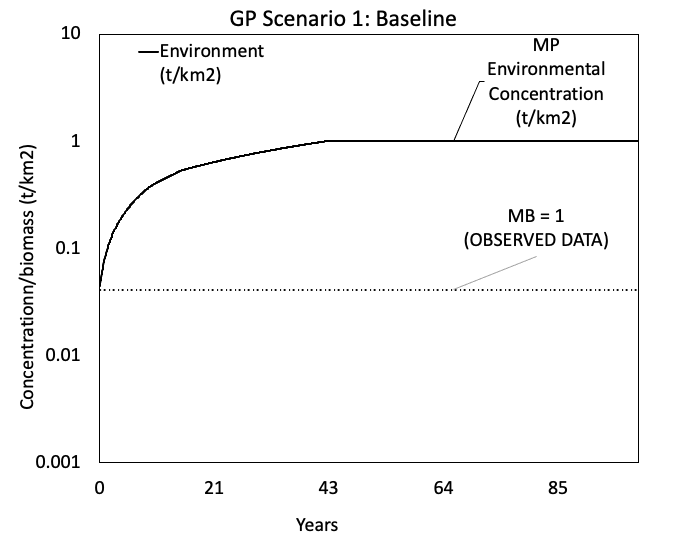 | 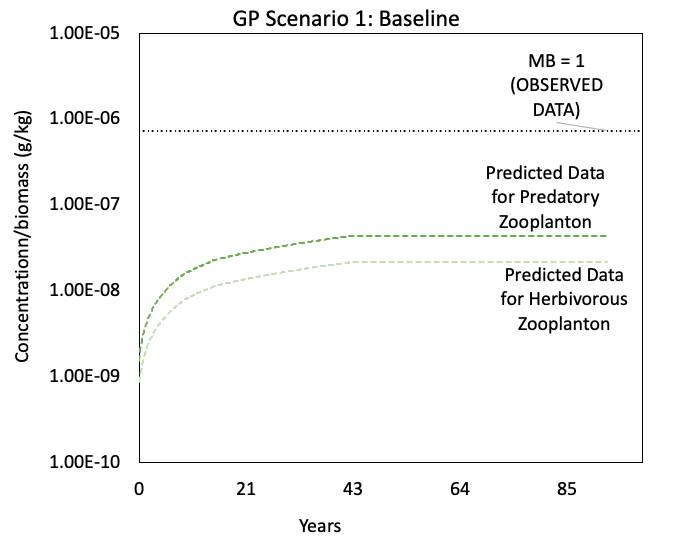 |
| --- | --- |
| 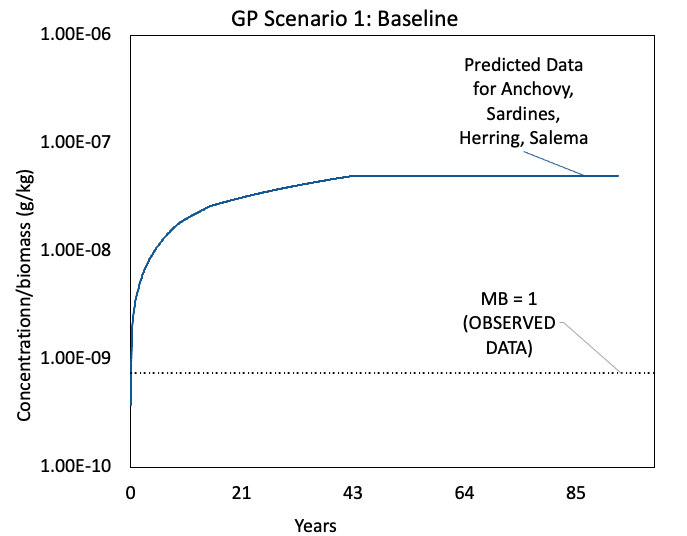 | 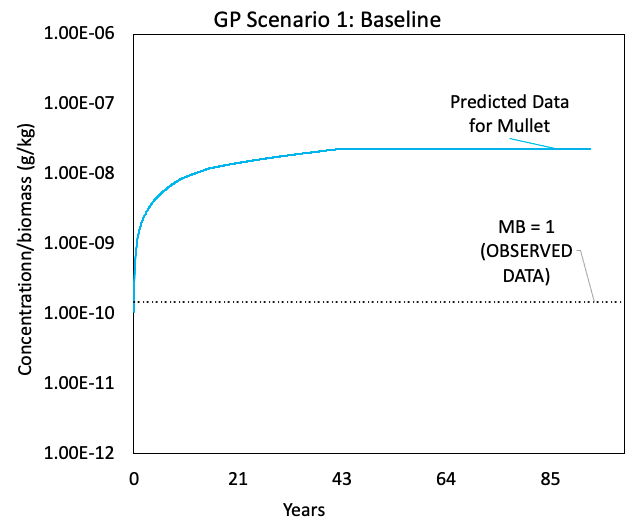 |

B)

| 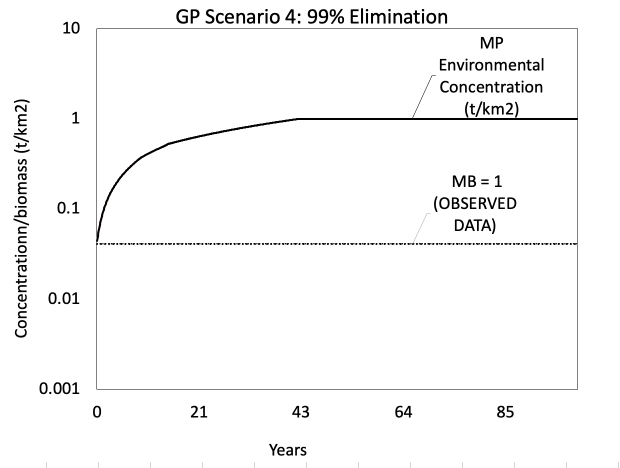 | 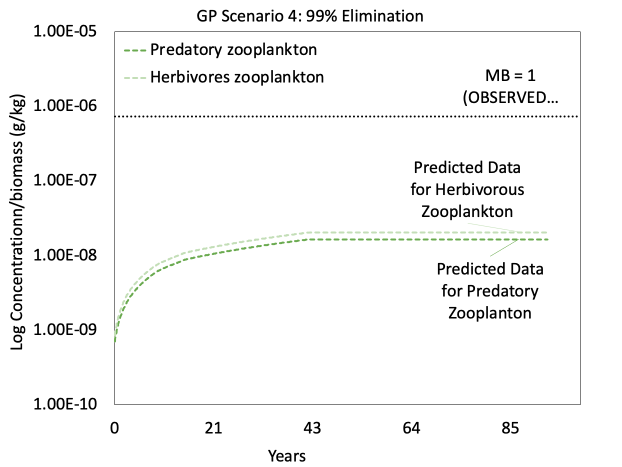 |
| --- | --- |
| 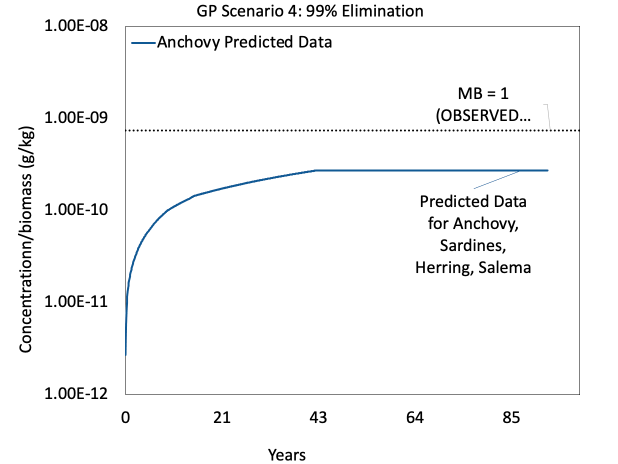 | 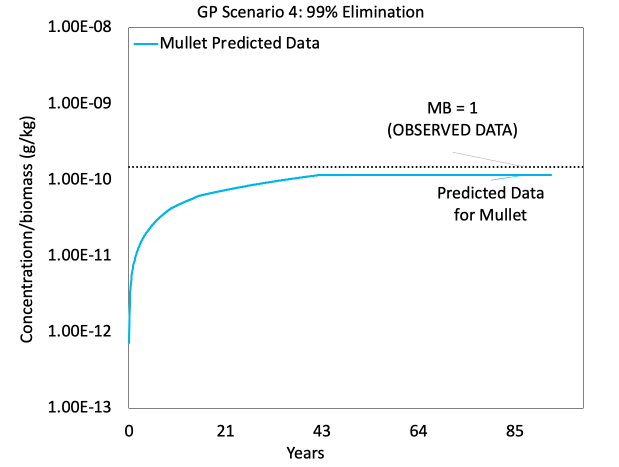 |

C)

| 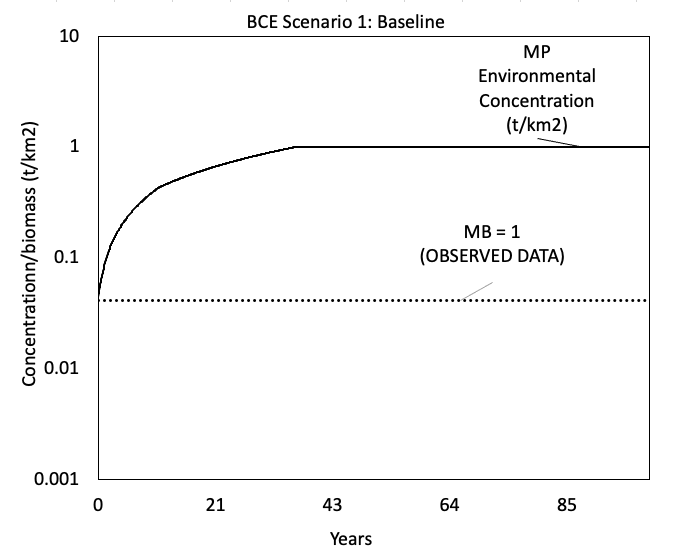 | 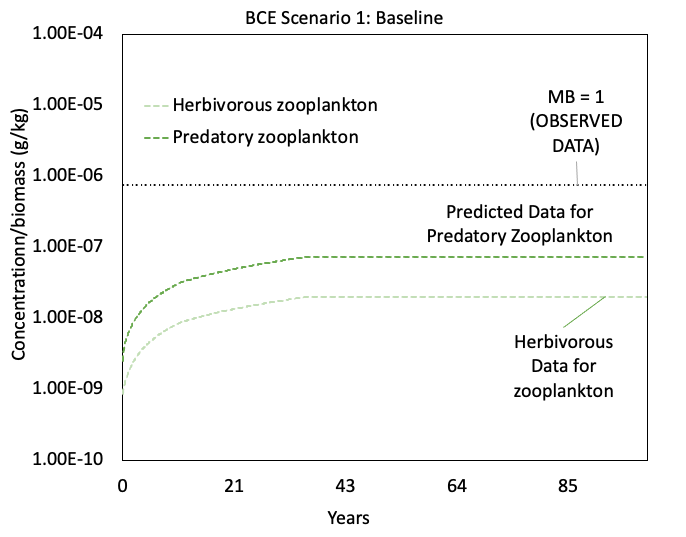 |
| --- | --- |
| 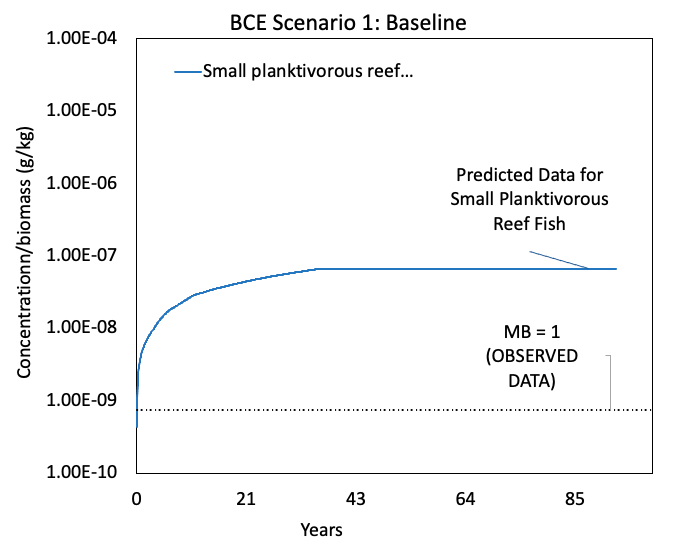 | 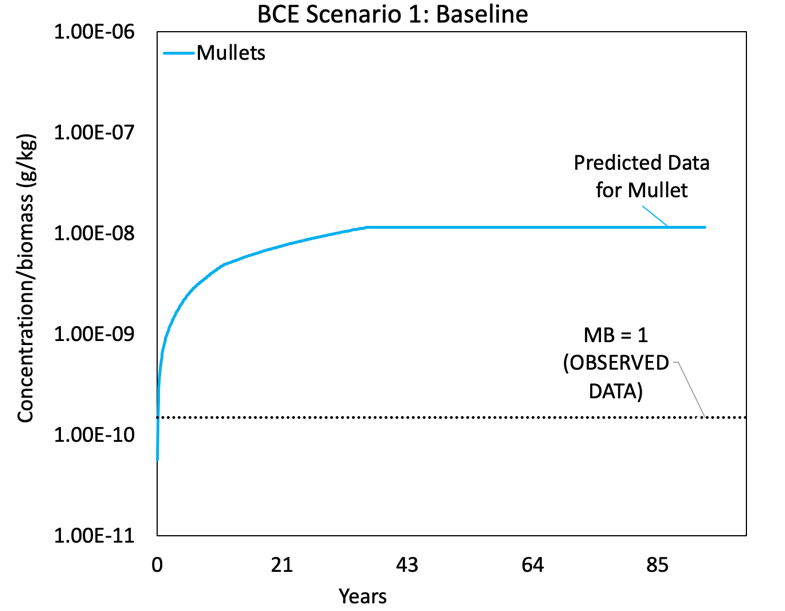 |

D)

| 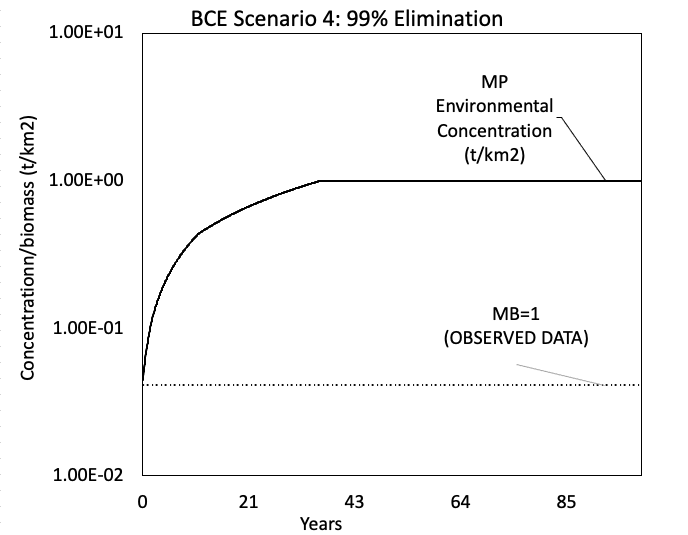 | 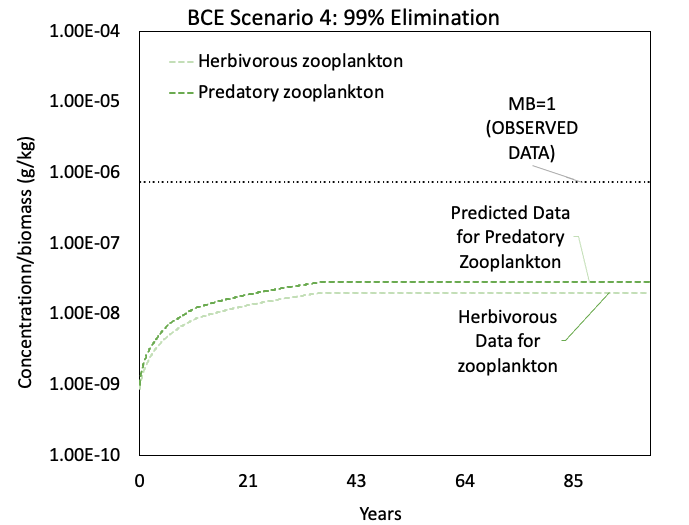 |
| --- | --- |
| 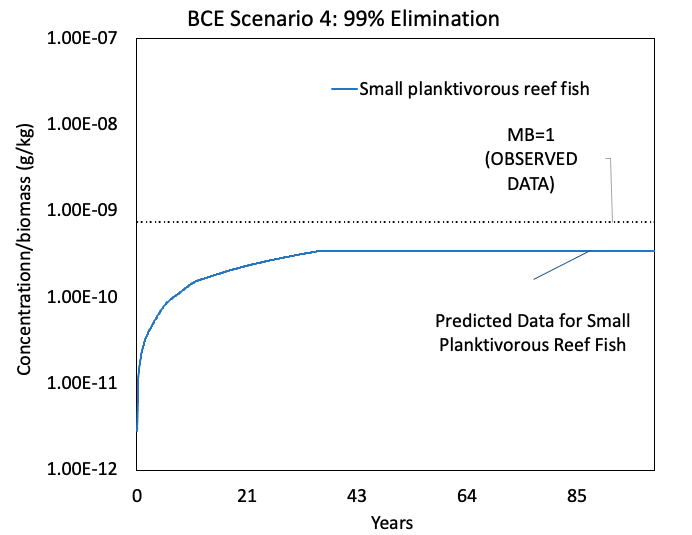 | 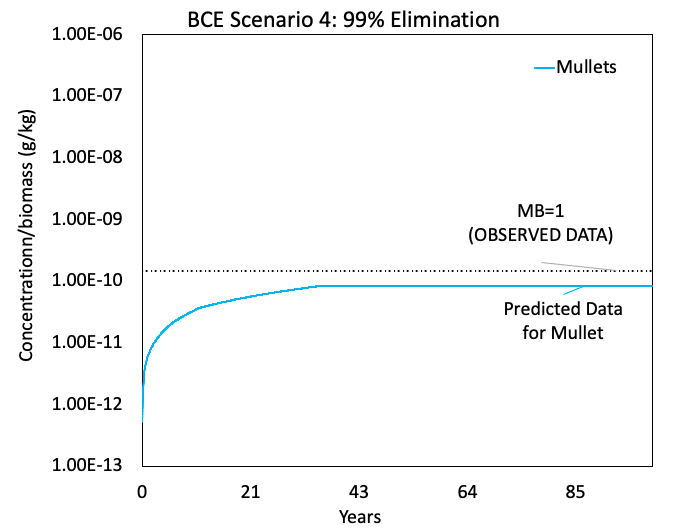 |

**Supplementary Figure S1:** Assessment of the model bias using the (A-B) Galápagos penguin (GP) and (C-D) Bolivar Channel Ecosystem (BCE) models run with Ecotracer to project microplastic concentration simulations from year 1 to 100 as predicted microplastic concentrations in the abiotic and biotic compartments. Results are presented by seawater microplastic abundance, microplastic concentrations in zooplankton, microplastic concentrations in anchovies, and microplastic concentrations in mullets. Predicted data is compared to empirically measured data on anthropogenic particle concentrations in Galápagos seawater and ingestion rates for zooplankton, anchovies, and mullets collected in October 2021. Two scenarios are presented for each model: GP model baseline (A), GP 99% elimination rates (B), BCE model baseline (C), BCE 99% elimination rates (D).

**References**

See references in the main text.
