# Supplementary material for: Modelling microplastic bioaccumulation and biomagnification potential in the Galápagos penguin ecosystem using Ecopath and Ecosim (EwE) with Ecotracer
Source: PLoS One. 2024 Jan 24;19(1):e0296788. doi: 10.1371/journal.pone.0296788 (PMC10807758; doi:10.1371/journal.pone.0296788)
Supplement: Attachment — Submitted filename: Response to Reviewers.docx [file pone.0296788.s002.docx]

**Response to Reviewers**

| **Editor Comments** | **Authors’ Response** |
| --- | --- |
| 1. Please ensure that your manuscript meets PLOS ONE's style requirements, including those for file naming. The PLOS ONE style templates can be found at  https://journals.plos.org/plosone/s/file?id=wjVg/PLOSOne_formatting_sample_main_body.pdf and  https://journals.plos.org/plosone/s/file?id=ba62/PLOSOne_formatting_sample_title_authors_affiliations.pdf | The manuscript and supplementary information have been reformatted in accordance with PLOS ONE’s style requirements. |
| 2. Note from Emily Chenette, Editor in Chief of PLOS ONE, and Iain Hrynaszkiewicz, Director of Open Research Solutions at PLOS: Did you know that depositing data in a repository is associated with up to a 25% citation advantage (https://doi.org/10.1371/journal.pone.0230416)? If you’ve not already done so, consider depositing your raw data in a repository to ensure your work is read, appreciated and cited by the largest possible audience. You’ll also earn an Accessible Data icon on your published paper if you deposit your data in any participating repository (https://plos.org/open-science/open-data/#accessible-data). | We have successfully uploaded our metadata to Sea Scientific Open Data Publication (SEANOE https://www.seanoe.org/) and included the citation in the main manuscript.  The Data Availability statement can now reflect that all underlying data can be accessed via SEANOE.  McMullen K, Hernán VF, Calle P, Alavarado-Cadena O, Pakhomov E, Alava JJ. Modelling microplastic bioaccumulation and biomagnification potential in the Galápagos penguin ecosystem using Ecopath and Ecosim (EwE) with Ecotracer. SEANOE. 2023. https://doi.org/10.17882/97201 |
| 3. We note that the grant information you provided in the ‘Funding Information’ and ‘Financial Disclosure’ sections do not match.  When you resubmit, please ensure that you provide the correct grant numbers for the awards you received for your study in the ‘Funding Information’ section. | We have rectified the funding information as per your guidelines. The funding statement now stands as follows:  "This research was partially funded by the Nippon Foundation via the Nippon Foundation-Marine Litter Project at the Institute for the Oceans and Fisheries, University of British Columbia, awarded to J. J. Alava and E. A. Pakhomov under award number F19-02677 (NIPPFOUN 2019) in collaboration with and via the Nippon Foundation-Ocean Nexus Center (Dr. Yoshitaka Ota) at University of Washington [https://oceannexus.uw.edu/about/]. This research was partially supported by the NSERC Discovery Grant RGPIN-2014-05107 to E. A. Pakhomov [https://www.nserc-crsng.gc.ca/professors-professeurs/grants-subs/dgigp-psigp_eng.asp]. Both the Nippon Foundation and NSERC funders had no involvement in the study design, data collection and analysis, decision to publish, or preparation of the manuscript." |
| 4. Thank you for stating the following in the Acknowledgments Section of your manuscript:  [A special thank you to V. Christensen and S. De la Puente Jeri for the FISH 501, an ecosystem modelling course carried out in 2021, which included phenomenal instruction and support. Authors thank and acknowledge the creators of the Bolivar Channel EwE Model, D. J. Ruiz and M. Wolff. A special thank you to D. J. Ruiz, who provided access and support throughout. Access to D. J. Ruiz and M. Wolff’s model made this research more robust. Thank you for your data transparency. Thank you to B. Hunt for offering constructive comments and feedback. K. McMullen and J. J. Alava thank the Nippon Foundation for providing funding to support fieldwork and research in Ecuador via the Nippon Foundation-Marine Litter Project at the Institute for the Oceans and Fisheries, University of British Columbia. Dr. Wilf Swartz proactively helped to manage funding and financial resources allocation and administration to support the Nippon Foundation-Marine Litter Project. This research was partially supported by the NSERC Discovery Grant RGPIN-2014-05107 to E.A.P. We humbly express our gratitude to the local people of the Galápagos Islands and the unique endemic and native species of the archipelago, who continue to inspire our desire for conservation.]  We note that you have provided funding information that is not currently declared in your Funding Statement. However, funding information should not appear in the Acknowledgments section or other areas of your manuscript. We will only publish funding information present in the Funding Statement section of the online submission form.  Please remove any funding-related text from the manuscript and let us know how you would like to update your Funding Statement. Currently, your Funding Statement reads as follows:  [This research was partially funded by the Nippon Foundation via the Nippon Foundation-Marine Litter Project at the Institute for the Oceans and Fisheries, University of British Columbia, awarded to J. J. Alava. https://oceannexus.uw.edu/about/  This research was partially supported by the NSERC Discovery Grant RGPIN-2014-05107 to E. A. Pakhomov. https://www.nserc-crsng.gc.ca/professors-professeurs/grants-subs/dgigp-psigp_eng.asp  The funders had no role in study design, data collection and analysis, decision to publish, or preparation of the manuscript.]  Please include your amended statements within your cover letter; we will change the online submission form on your behalf. | We have rectified the funding information as per your guidelines. The funding statement now stands as follows:  "This research was partially funded by the Nippon Foundation via the Nippon Foundation-Marine Litter Project at the Institute for the Oceans and Fisheries, University of British Columbia, awarded to J. J. Alava and E. A. Pakhomov under award number F19-02677 (NIPPFOUN 2019) in collaboration with and via the Nippon Foundation-Ocean Nexus Center (Dr. Yoshitaka Ota) at University of Washington [https://oceannexus.uw.edu/about/]. This research was partially supported by the NSERC Discovery Grant RGPIN-2014-05107 to E. A. Pakhomov [https://www.nserc-crsng.gc.ca/professors-professeurs/grants-subs/dgigp-psigp_eng.asp]. Both the Nippon Foundation and NSERC funders had no involvement in the study design, data collection and analysis, decision to publish, or preparation of the manuscript."  We have removed the funding information from the acknowledgment section, which now reads:  [A special thank you to V. Christensen and S. De la Puente Jeri for the FISH 501, an ecosystem modelling course carried out in 2021, which included phenomenal instruction and support. Authors thank and acknowledge the creators of the Bolivar Channel EwE Model, D. J. Ruiz and M. Wolff. A special thank you to D. J. Ruiz, who provided access and support throughout. Access to D. J. Ruiz and M. Wolff’s model made this research more robust. Thank you for your data transparency. Thank you to B. Hunt for offering constructive comments and feedback. Dr. Wilf Swartz proactively helped to manage funding and financial resources allocation. Thank you to the Galapagos National Park (research permit PC-92-22). We humbly express our gratitude to the local people of the Galápagos Islands and the unique endemic and native species of the archipelago, who continue to inspire our desire for conservation.] |
| 5. In your Data Availability statement, you have not specified where the minimal data set underlying the results described in your manuscript can be found. PLOS defines a study's minimal data set as the underlying data used to reach the conclusions drawn in the manuscript and any additional data required to replicate the reported study findings in their entirety. All PLOS journals require that the minimal data set be made fully available. For more information about our data policy, please see http://journals.plos.org/plosone/s/data-availability.  Upon re-submitting your revised manuscript, please upload your study’s minimal underlying data set as either Supporting Information files or to a stable, public repository and include the relevant URLs, DOIs, or accession numbers within your revised cover letter. For a list of acceptable repositories, please see http://journals.plos.org/plosone/s/data-availability#loc-recommended-repositories. Any potentially identifying patient information must be fully anonymized.  Important: If there are ethical or legal restrictions to sharing your data publicly, please explain these restrictions in detail. Please see our guidelines for more information on what we consider unacceptable restrictions to publicly sharing data: http://journals.plos.org/plosone/s/data-availability#loc-unacceptable-data-access-restrictions. Note that it is not acceptable for the authors to be the sole named individuals responsible for ensuring data access.  We will update your Data Availability statement to reflect the information you provide in your cover letter. | We have successfully uploaded our metadata to Sea Scientific Open Data Publication (SEANOE https://www.seanoe.org/) and included the citation in the main manuscript.  The Data Availability statement can now reflect that all underlying data can be accessed via SEANOE.  McMullen K, Hernán VF, Calle P, Alavarado-Cadena O, Pakhomov E, Alava JJ. Modelling microplastic bioaccumulation and biomagnification potential in the Galápagos penguin ecosystem using Ecopath and Ecosim (EwE) with Ecotracer. SEANOE. 2023. https://doi.org/10.17882/97201 |
| 6. We note that you have indicated that data from this study are available upon request. PLOS only allows data to be available upon request if there are legal or ethical restrictions on sharing data publicly. For more information on unacceptable data access restrictions, please see http://journals.plos.org/plosone/s/data-availability#loc-unacceptable-data-access-restrictions.  In your revised cover letter, please address the following prompts:  a) If there are ethical or legal restrictions on sharing a de-identified data set, please explain them in detail (e.g., data contain potentially sensitive information, data are owned by a third-party organization, etc.) and who has imposed them (e.g., an ethics committee). Please also provide contact information for a data access committee, ethics committee, or other institutional body to which data requests may be sent.  b) If there are no restrictions, please upload the minimal anonymized data set necessary to replicate your study findings as either Supporting Information files or to a stable, public repository and provide us with the relevant URLs, DOIs, or accession numbers. For a list of acceptable repositories, please see http://journals.plos.org/plosone/s/data-availability#loc-recommended-repositories.  We will update your Data Availability statement on your behalf to reflect the information you provide. | We have successfully uploaded our metadata to Sea Scientific Open Data Publication (SEANOE https://www.seanoe.org/) and included the citation in the main manuscript.  The Data Availability statement can now reflect that all underlying data can be accessed via SEANOE.  McMullen K, Hernán VF, Calle P, Alavarado-Cadena O, Pakhomov E, Alava JJ. Modelling microplastic bioaccumulation and biomagnification potential in the Galápagos penguin ecosystem using Ecopath and Ecosim (EwE) with Ecotracer. SEANOE. 2023. https://doi.org/10.17882/97201 |
| 7. We noted in your submission details that a portion of your manuscript may have been presented or published elsewhere. [Data from this article is published in K. McMullen master's thesis at the University of British Columbia, Institute for Oceans and Fisheries. It has not been published elsewhere.] Please clarify whether this [conference proceeding or publication] was peer-reviewed and formally published. If this work was previously peer-reviewed and published, in the cover letter please provide the reason that this work does not constitute dual publication and should be included in the current manuscript. | We would like to clarify that this data has not been published in scientific journals. The University of British Columbia published it as part of K. McMullen’s master’s thesis dissertation. As is common practice, the master’s thesis data and text have been revised and adjusted for this publication. The thesis reference is:  McMullen, K. (2023). The Galápagos penguin as the "canary in the coal mine" for microplastics research in the Galápagos Marine Reserve & plastic pollution perceptions in Ecuadorian mangrove communities (Master’s dissertation, University of British Columbia). https://open.library.ubc.ca/soa/cIRcle/collections/ubctheses/24/items/1.0432346?o=0 |
| 8. We note that Figure 2A and 3A in your submission contain copyrighted images. All PLOS content is published under the Creative Commons Attribution License (CC BY 4.0), which means that the manuscript, images, and Supporting Information files will be freely available online, and any third party is permitted to access, download, copy, distribute, and use these materials in any way, even commercially, with proper attribution. For more information, see our copyright guidelines: http://journals.plos.org/plosone/s/licenses-and-copyright.  We require you to either (1) present written permission from the copyright holder to publish these figures specifically under the CC BY 4.0 license, or (2) remove the figures from your submission:  A. You may seek permission from the original copyright holder of Figure 2A and 3A to publish the content specifically under the CC BY 4.0 license.  We recommend that you contact the original copyright holder with the Content Permission Form (http://journals.plos.org/plosone/s/file?id=7c09/content-permission-form.pdf) and the following text:  “I request permission for the open-access journal PLOS ONE to publish XXX under the Creative Commons Attribution License (CCAL) CC BY 4.0 (http://creativecommons.org/licenses/by/4.0/). Please be aware that this license allows unrestricted use and distribution, even commercially, by third parties. Please reply and provide explicit written permission to publish XXX under a CC BY license and complete the attached form.”  Please upload the completed Content Permission Form or other proof of granted permissions as an ""Other"" file with your submission.  In the figure caption of the copyrighted figure, please include the following text: “Reprinted from [ref] under a CC BY license, with permission from [name of publisher], original copyright [original copyright year].”  B. If you are unable to obtain permission from the original copyright holder to publish these figures under the CC BY 4.0 license or if the copyright holder’s requirements are incompatible with the CC BY 4.0 license, please either i) remove the figure or ii) supply a replacement figure that complies with the CC BY 4.0 license. Please check copyright information on all replacement figures and update the figure caption with source information. If applicable, please specify in the figure caption text when a figure is similar but not identical to the original image and is therefore for illustrative purposes only. | We have replaced previously acquired images in Figures 2A and 3A with original illustrations created by K. McMullen's author to avoid copyright issues. |
| 9. Please include captions for your Supporting Information files at the end of your manuscript, and update any in-text citations to match accordingly. Please see our Supporting Information guidelines for more information: http://journals.plos.org/plosone/s/supporting-information. | We have included captions and the appropriate text to identify supplementary information tables and figures as outlined in the PLOS ONE guidelines. Namely, we have added an "s" preceding the table or figure number. |
| 10. Please review your reference list to ensure that it is complete and correct. If you have cited papers that have been retracted, please include the rationale for doing so in the manuscript text, or remove these references and replace them with relevant current references. Any changes to the reference list should be mentioned in the rebuttal letter that accompanies your revised manuscript. If you need to cite a retracted article, indicate the article’s retracted status in the References list and also include a citation and full reference for the retraction notice. | The reference list has been thoroughly revised as per the modifications outlined in the track changes file provided. |
| **Reviewers’ Comments** | **Authors’ Response** |
| 1. Is the manuscript technically sound, and do the data support the conclusions?  The manuscript must describe a technically sound piece of scientific research with data that supports the conclusions. Experiments must have been conducted rigorously, with appropriate controls, replication, and sample sizes. The conclusions must be drawn appropriately based on the data presented.  Reviewer #1: Yes  Reviewer #2: Yes | NA |
| 2. Has the statistical analysis been performed appropriately and rigorously?  Reviewer #1: Yes  Reviewer #2: Yes  3. Have the authors made all data underlying the findings in their manuscript fully available?  The [PLOS Data policy](http://www.plosone.org/static/policies.action#sharing) requires authors to make all data underlying the findings described in their manuscript fully available without restriction, with rare exception (please refer to the Data Availability Statement in the manuscript PDF file). The data should be provided as part of the manuscript or its supporting information, or deposited to a public repository. For example, in addition to summary statistics, the data points behind means, medians and variance measures should be available. If there are restrictions on publicly sharing data—e.g. participant privacy or use of data from a third party—those must be specified.  Reviewer #1: Yes  Reviewer #2: Yes | NA |
| 4. Is the manuscript presented in an intelligible fashion and written in standard English?  PLOS ONE does not copyedit accepted manuscripts, so the language in submitted articles must be clear, correct, and unambiguous. Any typographical or grammatical errors should be corrected at revision, so please note any specific errors here.  Reviewer #1: Yes  Reviewer #2: Yes | NA |
| 5. Review Comments to the Author  Please use the space provided to explain your answers to the questions above. You may also include additional comments for the author, including concerns about dual publication, research ethics, or publication ethics. (Please upload your review as an attachment if it exceeds 20,000 characters)  Reviewer #1: The manuscript entitled “Modelling microplastic bioaccumulation and biomagnification potential in the Galápagos penguin ecosystem using Ecopath and Ecosim (EwE) with Ecotracer” is an interesting piece of work and provides a new direction to research. There are certain points to be considered by the authors. Hence it is suggest for Minor Revision.   1. The term microplastics is not defined at the first instance in the introduction. 2. The overall manuscript is well written. 3. The methodology is clearly written, however is very lengthy. Some information may be shifted to the supplementary section. 4. Results section is very lengthy and loses focus of the primary aim of the article. 5. There are numerous tables, some of which could be shifted to the supplementary material. 6. Some figures have been misquoted. For example, Figure 16 A, Figure 18 B. However, such figures are not provided in the manuscript.   The manuscript can be accepted for publication after these minor revisions. | Regarding the reviewers' comments, we have read and addressed each point they raised:   1. The definition of "microplastics" has been incorporated in the introduction paragraph. 2. NA 3. We have reduced the methodology, shifting some details to the supplementary section, but we would like to highlight that we retained portions of the Galapagos penguin model methods; we feel that because the Galapagos penguin model is novel, data inputs need to be justified and explained thoroughly in the main manuscript. This differs from the Bolivar Channel, in which the methodology is already extensively outlined in Ruiz & Wolff (54). 4. We have streamlined the results section to center on the primary aim of the article, but again, we wish to point out that the Galapagos penguin model is novel. Thus, results must indicate the results of the model itself and then the bioaccumulation and biomagification results using Ecotracer. This, again, is different from the Boliver Channel model, in which the general model results were previously published in Ruiz & Wolff (54). 5. We have reduced the number of tables from 13 to 7, shifting the excluded tables to supplementary information. 6. We rectified discrepancies in figure labels and tables, ensuring alignment with the guidelines provided.   Thank you kindly for the revisions and considering our article. We appreciate you time. |
| Reviewer #2: No Comments required because they organized everything very clearly. This manuscript is very interesting and it will be helpful to the science community. The authors mention everything according to the aim and objective. | Thank you kindly for this positive feedback. We appreciate it and extend our gratitude to you for taking the time to review. |
| 6. PLOS authors have the option to publish the peer review history of their article ([what does this mean?](https://journals.plos.org/plosone/s/editorial-and-peer-review-process#loc-peer-review-history)). If published, this will include your full peer review and any attached files.  If you choose “no”, your identity will remain anonymous but your review may still be made public.  **Do you want your identity to be public for this peer review?** For information about this choice, including consent withdrawal, please see our [Privacy Policy](https://www.plos.org/privacy-policy).  Reviewer #1: No  Reviewer #2: No | NA |
